# The effect of instability resistance training on balance ability among athletes: a systematic review

**DOI:** 10.3389/fphys.2024.1434918

**Published:** 2025-01-07

**Authors:** Jianxin Gao, Xinxin Fu, Hang Xu, Qi Guo, Xiaopeng Wang

**Affiliations:** ^1^ College of Physical Culture, Jiangxi Teachers College, Yingtan, Jiangxi, China; ^2^ Department of Sport Studies, Faculty of Educational Studies, University Putra Malaysia, Serdang, Selangor, Malaysia

**Keywords:** athletes, balance ability, BOSU and Swiss ball, instability resistance training, unstable environment

## Abstract

**Background:**

Instability resistance training (IRT) has been the focus of extensive research because of its proven benefits to balance ability, core stability, and sports performance for athletes. However, there is a lack of systematic reviews explicitly evaluating IRT’s impact on athletes’ balance ability. This study aims to conduct a systematic review of the effects of IRT on balance ability among athletes.

**Method:**

This study used guidelines for the systematic review and meta-analysis of PRISMA, Web of Science, EBSCOhost (SPORTDiscus), PubMed, Scopus, and Google Scholar to collect original references in electronic databases. The PICOS method was selected for the inclusion criteria. The physiotherapy evidence database (PEDro) scale was used to assess the scoring for articles’ risk range of bias. The scoring of 20 studies ranges from 4–8, and study quality is moderate to high.

**Results:**

Out of 285 identified studies, only 20 articles fulfilled all the eligibility criteria after screening. IRT could significantly improve reciprocal, static, and dynamic balance ability among judo athletes, basketball players, weightlifters, archery athletes, soccer players, rhythmic gymnasts, badminton players, track and field athletes, handball players, volleyball players, and gymnasts using unstable surfaces or environments (i.e., BOSU, Swiss, Wobble boards, Suspension trainer, Sissel pillows, Inflated disc and foam surface, Airex balance pad, Togu power ball, Thera-Band, Elastic band strap, Sand surface and so on).

**Conclusion:**

The finding suggests that different types of IRT benefit athletes as this training method can effectively enhance reciprocal, static, and dynamic balance ability in athletes. Therefore, this review suggests that IRT should be considered in athletes’ daily training routines for the physical fitness of reciprocal, static, and dynamic balance ability.

## 1 Introduction

Balance refers to the ability of the human body to automatically adjust and maintain a posture when subjected to external forces during rest or movement (Wulf and Lewthwaite, 2009; Zhu et al., 2014; Bliss and Teeple, 2005). In addition, balance is a fundamental condition for completing various sports skills such as athletics, ice skating, swimming, ball games, gymnastics, dance, martial arts, etc. The ability to balance is not only related to the integrity and symmetry of the body structure but also closely related to the vestibular organs, visual organs, and proprioception of the human body (Wei et al., 2016). These three systems form the triple system of balance in the human body (i.e., the mutual correlation and close relationship between brain balance regulation, cerebellar ataxia coordination, and limb muscle strength) (Tayshete et al., 2020). So, the balance depends on the comprehensive coordination ability of the human body to stimuli from vestibular organs and proprioceptive receptors composed of muscles, tendons, joints, and visual organs.

Balance training has attracted the attention of many athletes, coaches, sports workers, and researchers. In improving athletic performance, balance ability can help athletes better unleash their physical potential (Bouteraa et al., 2020). The coordinated operation of various parts of an athlete is critical to achieving various sports performances. Balance can improve an athlete’s flexibility and agility, enabling them to maintain balance in multiple parts, coordinate and react quickly, adjust body posture during exercise, promote smooth technical movements, and maximize strength and quality (Zech et al., 2010). Moreover, external motivational stimuli, such as music or verbal encouragement, have been shown to improve task performance significantly, particularly in untrained individuals. For example, endurance tasks performed under musical stimulation or verbal encouragement demonstrated increased task duration by over 15% compared to standard conditions. This enhancement is attributed to more effective motor unit recruitment, as evidenced by reduced electromyographic trend coefficients over time, suggesting greater neuromuscular efficiency (Biceps Brachii: −10.39%, Brachioradialis: −9.40%, p < 0.001) (Cotellessa et at., 2024). Integrating balance training with motivational stimuli could further amplify an athlete’s ability to maintain posture, execute precise technical movements, and delay fatigue, fostering an environment that supports optimal performance outcomes. In terms of improving sports’ competitive ability, balance ability helps athletes develop other physical fitness such as strength, speed, endurance, agility, coordination, etc., reasonably and comprehensively, reducing fatigue and allowing athletes to utilize their strength in training and competition fully (Gebel et al., 2020; Zemková, 2014). Poor body posture and uneven distribution of muscle strength can easily lead to excessive force on certain parts of athletes, resulting in sports injuries (Hrysomallis, 2007). In terms of preventing sports injuries, balance ability can improve the adaptability of athletes’ body posture during exercise, provide better support and protection for joints and muscles, and minimize and reduce sports injuries and risks in high-intensity sports for athletes (Chang et al., 2020). In terms of promoting athlete health, good balance ability is not only beneficial for physical health during sports. Still, it can also improve the athlete’s mental state, reduce anxiety and stress, promote physical and psychological balance, and enhance their confidence and emotional regulation ability (Schaal et al., 2011; Whitehead and Senecal, 2020).

To improve the balance ability of athletes, traditional resistance training (TRT) methods were used (Kibele and Behm, 2009; Šarabon and Kozinc, 2020). The advantage of the TRT effect is that it could significantly improve the development of enormous muscle group strength for proprioception and help refine and standardize athletes’ balance ability and motor skills (Yu et al., 2008). However, TRT methods have two common disadvantages. The first is the stable training state or environment with relative support provided by the instrument or ground for TRT on land (Winwood et al., 2015). The second is in the process of TRT; the athlete’s center of gravity is completed in a relatively stable state, and the athlete’s muscle is fixed with one end for concentric isotonic training (Krzysztofik et al., 2019). As a consequence, the limitation of TRT is that the small muscles in the deep layer of the athletes (coordination between agonistic and antagonistic muscles) are not well recruited and activated. Nevertheless, these muscles play a crucial role in the balance ability of the human body (Sriwarno et al., 2008). Combining the above, although the TRT method can improve the balance ability of sports athletes to a certain extent, there must be a more effective training method. So, to improve the balance ability of athletes, the TRT method cannot be blindly used. Coaches can try new unstable training methods that can produce an “unstable training effect” rather than a “stable training effect” to improve athletes’ balance ability (Gao et al., 2023).

Instability resistance training (IRT) is widely used in sports rehabilitation and physical fitness training. The main characteristic that distinguishes it from TRT is unstable conditions, surfaces, and environments (including specific equipment) (Norwood et al., 2007; Mehrpuya and Moghadasi, 2019). Another main difference is the load, since while TRT involves considerable loads, in IRT, given that there is instability, the loads must be reduced. Unstable surfaces, platforms, and environments can be built with professional equipment, such as Wobble Boards, Swiss or BOSU, suspended chains, foam rollers, and bands. In addition, unstable training conditions can use snow, water, sand, gravel materials, and so on (Colorado et al., 2020). The reasons for choosing IRT for athletes are as follows. Firstly, IRT is an advanced training stage that theoretically enhances muscle strength and balance ability to achieve core stability. This IRT method can activate and recruit deep and minor muscle groups and obtain proprioceptive neuromuscular sensation, thereby further enhancing the mobilization of muscle strength and balance ability (Mehrpuya and Moghadasi, 2019). Secondly, regarding unstable surfaces and environments, IRT could provide instruments for unstable surfaces or environments (For example, swing boards, Swiss, BOSU, elastic and suspension devices). Therefore, an unstable environment can not only provide athletes with instability loads and simulation to the maximum extent possible (Sekendiz et al., 2010; Cuğ et al., 2012; Xu et al., 2020).

Most studies have mentioned IRT’s efficiency for athletes’ balance ability. However, the IRT training method must be systematically sorted out in terms of its structural function and theoretical and practical application. With a relatively short history, a systematic review of the effectiveness of varying degrees of IRT intervention is still lacking. Therefore, this study aims to systematically review the current articles on IRT’s impact on athletes’ balance ability.

## 2 Materials and methods

### 2.1 Registration on INPLASY

The registration on the International Platform of Registered Systematic Review and Meta-analysis Protocols (INPLASY), the study was assigned the registration number INPLASY2023100048, with a corresponding DOI number of 10.37766/inplasy 2023.10.0048, accessible on the platform’s website: https://inplasy.com/.

### 2.2 Databases and keywords

This article used the following databases: Web of Science, EBSCOhost (SPORT Discussion), PubMed, Scopus, Google Scholar, and References until the end of 2023. The keywords in this study were: (“instability resistance training” OR “instability resistance exercise” OR “unstable surface training” OR “unstable training” OR “unstable surface exercise” OR “unstable exercise”) AND (“balance” OR “balance ability” OR “reciprocal balance” OR “dynamic balance” OR “static balance” OR “reciprocal balance ability” OR “dynamic balance ability” OR “static balance ability”) AND (“player” OR “athlete” OR “sportsman” OR “sportswoman” OR “sportsperson”).

### 2.3 Eligibility criteria

The PICOS model was used in this study. This model has five parts of the population: intervention, comparison, outcome, and study design. The details of the inclusion criteria for the five parts are shown in Table 1.

**TABLE 1:** Inclusion and eligibility criteria.

PICOS	Detailed information on inclusion and eligibility criteria
Population	Healthy athletes or players cannot distinguish between age and gender
Intervention	IRT (with the different unstable surfaces), IRT+, another training format in, E.G., (not <4 weeks)
Comparison	Single (within group), multiple-group trials (between groups)
Outcome	The outcome must comprise the impact of IRT on balance ability among athletes and players
Study Design	Single-group and randomized controlled trials (RCT or CRCT)

### 2.4 Search, screening, and selection processes

Firstly, this article used the EndNote X8 citation management system to eliminate duplicate articles. Secondly, the authors conducted a first round of screening on the literature that met the requirements based on the title and abstract. Then, according to the complete text, they conducted a second screening round on the literature that had already been selected in the first round. Thirdly, literature that meets the standards was evaluated to determine the final reliability literature. Finally, at the seminar, all authors reached a consensus on which literature would be most selected for systematic review.

### 2.5 Data extraction and PEDro scale assessment

Based on reading the entire literature, the authors summarized the following content using standardized templates. In addition, this study used the PEDro scale (Verhagen et al., 1998), which has been proven reliable when building system reviews. PEDro scale is a Delphi series of scales specifically developed by Verhagen and colleagues for epidemiology. The same authors performed the statistical analysis.

## 3 Results

### 3.1 Article selection

**FIGURE 1:**
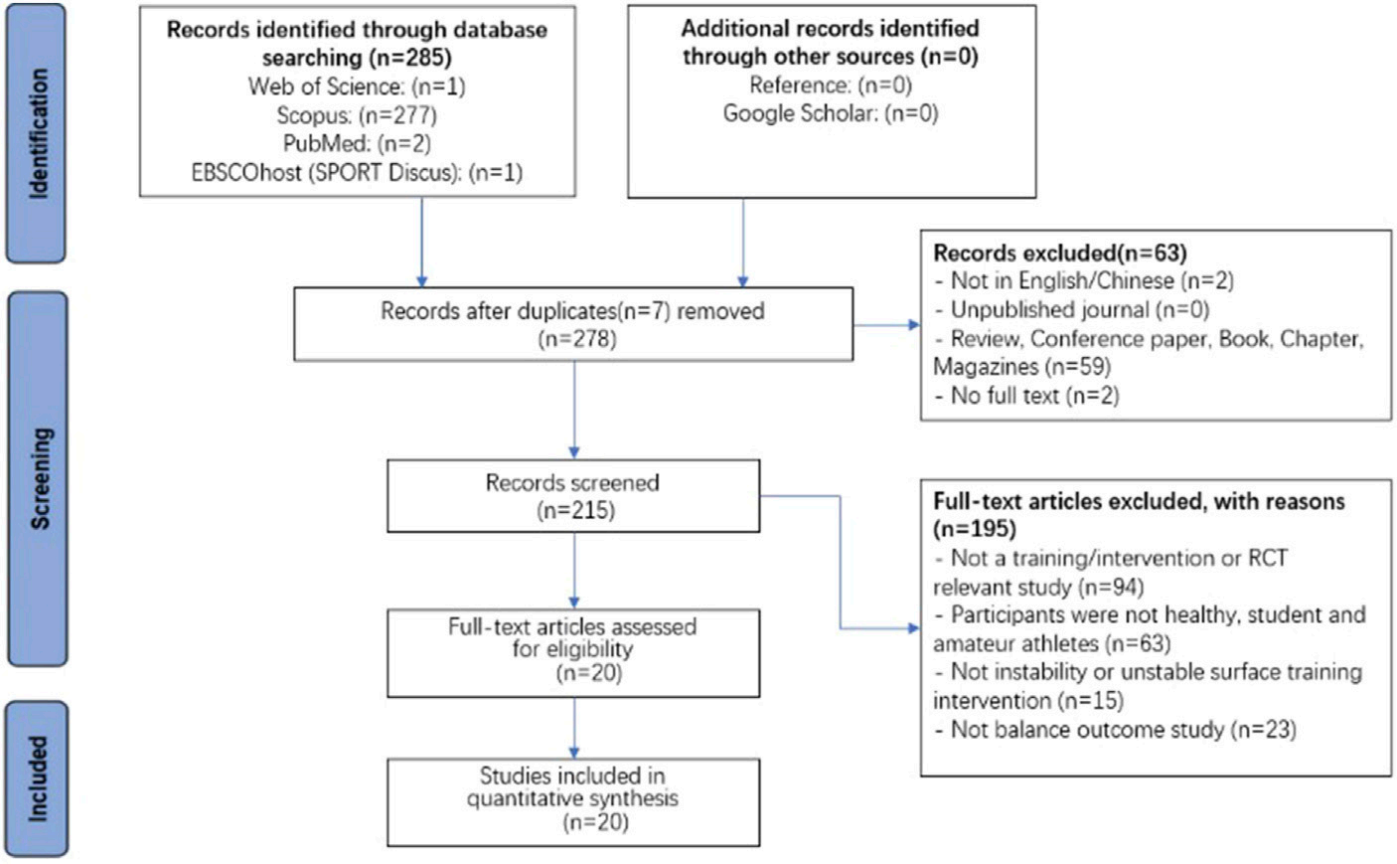
The identification, screening, and included processes for articles based on PRISMA.

This study employed the methodology recommended by the Preferred Reporting Items for Systematic Reviews and Meta-Analyses (PRISMA) to systematically search, screen, and analyze pertinent data within the selected article. The procedure of identification, screening, and inclusion for the article is shown in Figure 1. Initially, the total number of 285 articles were the records identified through database searching. After preliminary removal of duplicate articles, there were 278 articles. After the first screening, 63 articles were removed, which included two articles not in English, 0 articles from unpublished journals, 59 articles reviewed, conference papers, books, chapters, and magazines, and two articles without full text. In the next screening phase, 215 eligibility articles were evaluated for full-text, 195 articles were removed, which included 94 articles that were not a training/intervention or RCT relevant study, 63 articles participants were not healthy, student and amateur athletes, 15 articles were not instability and unstable surface training intervention, and 23 articles were not balance outcome studies. In the end, there were only a total of 20 articles for quantitative synthesis that met the criteria. The detail is shown in Figure 1.

### 3.2 Study quality assessment

The PEDro scale includes 11 items to evaluate the quality of the method, with each item scoring only 0 and 1, with one representing yes and 0 representing no. If the literature score is higher, it indicates that the quality of the literature method is better. The quality valuation was interpreted as follows: 0–3 Points (poor), 4-5 points (moderate), 6–10 points (high). Table 2 shows the detailed scores in this study’s PEDro scale of 20 articles. In all studies with a score of 4-7, the study quality is moderate to high. The detail is shown in Table 2.

**TABLE 2:** Summary of PEDro scale assessment scores.

N	References	N1	N2	N3	N4	N5	N6	N7	N8	N9	N10	N11	Total PEDro Score	Study Quality
1	Norambuena et al. (2021)	1	0	0	1	0	0	0	1	0	0	1	4	Moderate
2	Thielen et al. (2020)	1	1	0	1	0	0	0	1	1	1	1	7	High
3	Kang et al. (2013)	1	1	0	1	0	0	0	1	1	1	1	7	High
4	Prasetyo et al. (2023)	1	0	0	1	0	0	0	1	0	0	1	4	Moderate
5	Makhlouf et al. (2018)	1	1	0	1	0	0	0	1	1	1	1	7	High
6	Cabrejas Mata et al. (2022)	0	1	0	1	0	0	0	1	1	1	1	6	Moderate
7	Zhao et al. (2021)	1	0	0	1	0	0	0	1	0	0	1	4	Moderate
8	Negra et al. (2017)	1	1	0	1	0	0	0	1	1	1	1	7	High
9	Romero-Franco et al. (2012)	1	1	0	1	0	0	0	1	1	1	1	7	High
10	Wee and Fatt (2019)	1	1	0	1	0	0	0	1	1	1	1	7	High
11	Hammami et al. (2022)	1	1	0	1	0	0	0	1	1	1	1	7	High
12	Hammami et al. (2023)	1	0	0	1	0	0	0	1	0	1	1	5	Moderate
13	Oliver and Brezzo (2009)	1	1	0	1	0	0	0	1	1	1	1	7	High
14	Gönener and Gönener (2020)	1	1	0	1	0	0	0	1	1	1	1	7	High
15	Zemková and Hamar (2010)	1	1	1	1	0	0	0	1	1	1	1	8	High
16	Hammami et al. (2020)	0	1	0	1	0	0	0	1	1	1	1	6	High
17	Eisen et al. (2010)	1	1	0	1	0	0	0	1	1	1	1	7	High
18	Gidu et al. (2022)	1	1	0	1	0	0	0	1	1	1	1	7	High
19	Granacher et al. (2015)	1	1	0	1	0	0	0	1	1	1	1	7	High
20	Chaouachi et al. (2017)	0	1	0	1	0	0	0	1	1	0	1	5	Moderate

Note: N1, eligibility criteria; N2, random alocation; N3, allocation concealment; N4, baseline comparability; N5, blind participants; N6, blind therapist; N7, Blind assessor; N8, Follow-Up; N9, intention to treat analysis; N10, group comparison; N11, point measure and variability. A detailed explanation for each PEDro scale item can be accessed at https://www.pedro.org.au/english/downloads/pedro-scale.

### 3.3 Participant characteristics

Table 3 shows the characteristics of participants, intervention, and outcomes for the 20 studies. 1) Classification by athletes. 6 articles were on soccer players (Makhlouf et al., 2018; Negra et al., 2017; Wee and Fatt, 2019; Gidu et al., 2022; Granacher et al., 2015; Chaouachi et al., 2017); basketball players of 2 articles (Thielen et al., 2020; Zemková and Hamar, 2010); handball players of 2 articles (Hammami et al., 2022; Hammami et al., 2020); weightlifters of 2 articles (Kang et al., 2013; Hammami et al., 2023), volleyball combined soccer players of 2 articles (Oliver and Brezzo, 2009; Eisen et al., 2010), judo athletes of 1 article (Norambuena et al., 2021), archery athletes of 1 article (Prasetyo et al., 2023), rhythmic gymnasts of 1 article (Cabrejas Mata et al., 2022), badminton players of 1 article (Zhao et al., 2021), including sprinters of 1 article (Romero-Franco et al., 2012), gymnasts of 1 article (Gönener and Gönener, 2020). 2) Participant, gender, and age. The total number of athletic subjects was 664 (459 males, 157 females, and 48 no reported gender). Among the 20 studies, 18 articles reported the classification of age means, including below 10 years old in 1 article (Gönener and Gönener, 2020), 10–15 years old in 9 articles (Kang et al., 2013; Makhlouf et al., 2018; Cabrejas Mata et al., 2022; Negra et al., 2017; Hammami et al., 2023; Gidu et al., 2022; Granacher et al., 2015; Chaouachi et al., 2017; Norambuena et al., 2021), 16–20 years old of 7 articles (Zhao et al., 2021; Thielen et al., 2020; Wee and Fatt, 2019; Hammami et al., 2022; Oliver and Brezzo, 2009; Zemková and Hamar, 2010; Hammami et al., 2020), above 20 years old of 1 article (Romero-Franco et al., 2012). However, 20 articles have already covered the age of 7–20 for young athletes, and a few of the 20 studies reported under 10 years old and above 20 years old. The detail is shown in Table 3.

**TABLE 3:** Participant, intervention, and main outcome for the 20 studies.

N	Study	Subjects	Intervention							Main outcome related to balance ability
			Type of athlete	Gender	Age	Type	Instability environment	Balance ability Measured index	Frequency and duration	
1	Norambuena et al. (2021)	10	Judo athletes	Mixed: 8 Female 2 Male	15.4 ± 2.8 years	E.G.,: Instability suspension training CG: No control group	1) Suspension trainer (TRX^®^, United States)	Reciprocal balance test: 1) Prone instability test Dynamic balance test: 1) Y balance test	5 times/Week, 5 weeks	E.G.,: Prone instability test↔ Y balance test of right leg↑, left leg↑, right arm↑, left arm↑
2	Thielen et al. (2020)	32	Basketball player	Male	20.4 ± 1.4 years	E.G.,: Suspended load program CG: Traditional barbell load program	1) The plates suspended from the barbell using non-elastic straps	Dynamic balance test: 1) Star excursion balance test (SEBT)	4 times/Week, 6 weeks	E.G., and CG: Star excursion balance test (SEBT)↔
3	Kang et al. (2013)	32	Weightlifter	Male	MS: 14y HS: 17y	E.G.,1 (MS): Instability balance training E.G.,2 (HS): Instability balance training CG1 (MS): No training CG2 (HS): No training	1) Swiss ball	Static balance test: 1) One-leg standing with closed eyes test	Unknown times/Week, 8 weeks	E.G.,2 (HS): One-leg standing time with eyes closed↑
4	Prasetyo et al. (2023)	12	Archery athletes	Unknown	14–17y	E.G.,: Instability suspension training CG: No control group	1) BOSU ball	Static balance test: 1) The stork stand test	18 circuit training sessions	E.G.,: Standing on one leg↑
5	Makhlouf et al. (2018)	57	Soccer player	Male	E.G.,1:11.06 ± 0.75y E.G.,2:11.29 ± 0.85y CG:10.98 ± 0.80y	E.G.,1 (BPT): Combined balance and plyometric training (instability training is a part of the program) E.G.,2 (APT): Agility-plyometric training (stable training) CG: Regular soccer training (stable training)	1)Swiss ball 2) An inflated disk 3) A foam surface progressing to a BOSU ball or inflated disk 4) Elastic band strap	Static balance test: 1) Standing stork test Dynamic balance test: 1) Y balance test	2 times/Week, 8 weeks	BTP: Standing stork (P< 0.01, d = 3.17) ↑, Y-balance (P< 0.01, d = 1.48) ↑ ATP: Standing stork (P< 0.01, d = 5.53) ↑, Y-balance (P< 0.01, d = 1.20) ↑ CG: Standing stork (P< 0.02, d = 1.30) ↑, Y-balance (*p* = 0.18, d = 0.28)↔
6	Cabrejas Mata et al. (2022)	44 E.G.,: 23 CG: 21	Rhythmic gymnast	Female	10.5 ± 1.8 years	E.G.,: Integrated functional core and plyometric training (instability training is a part of the program) CG: Usual training (stable training)	1) BOSU ball 2) Balance disks 3) Softballs	Static balance test: 1) Right/left support leg with eyes open test 2) Right/left support leg with eyes closed test	3 times/Week, 8 weeks	E.G.,: Right support leg with eyes open ↑; Left support leg with eyes open ↑
7	Zhao et al. (2021)	38	Badminton Player	Female	17 ± 1.1 y	E.G.,1 (HG): Integrative neuromuscular training (instability training is a part of the program) E.G.,1 (LG): Integrative neuromuscular training (instability training is a part of the program) CG: No control group	1) BOSU ball 2) Balance board 3) Swiss ball	Dynamic balance test: 1) Single-leg side hop test	4 times/Week, 8 weeks	E.G.,1 (HG): Single-leg side hop (L) ↑, Single-leg side hop (R) ↑ E.G.,2 (LG): Single-leg side hop (L) ↑, Single-leg side hop (R) ↑
8	Negra et al. (2017)	33	Soccer player	Male	PTS: 12.1 ± 0.5 years PTC: 12.2 ± 0.6 years	E.G.,: Unstable performed combined plyometric training (PTC) CG: Stable performed plyometric training (PTS)	1) Airex balance pad 2) Thera-Band stability trainer	Static balance test: 1) SSBT, stable stork balance test 2) USBT, unstable stork balance test	3–5 times/Week, 8 weeks	Between-group (post-test) SSBT↔, USBT ↑
9	Romero-Franco et al. (2012)	33	Sprinter	Male	21.82 ± 4.84 years	E.G.,: Proprioceptive training program on BOSU and Swiss ball CG: A shorter duration training	1) BOSU and Swiss ball	Reciprocal balance test: 1) Stability Test with Eyes Open and Closed; 2) Postural Stability; 3) Gravity Center Control	3 times/Week, 6 weeks	Between-group (post-test): XEO↑, Position of the gravity center in the posterior direction ↑, Position of the gravity center in the right direction ↑
10	Wee and Fatt (2019)	19	Soccer Player	Male	20 ± 1.73 years	E.G.,: Soccer program supplementary Swiss Ball Training (SBT) CG: Soccer program	1) Swiss Ball	Static balance test: 1) Standing Stork Test (SST)Dynamic balance test: 1) Four Step Square Test (FSST)	2 times/Week, 6 weeks	Between-group (post-test): SST↔, FSST↑
11	(M. Hammami et al., 2022)	42	Handball player	Male	E.G.,: 16.4 ± 0.4 years CG: 16.2 ± 0.4 years	E.G., (JSTG): Supplemental jump and sprint exercise training on sand CG: Standard in-season regimen	1) On sand	Static balance test: 1) Standing stork test Dynamic balance test: 2) Y balance test	3 times/Week, 7 weeks	E.G., (JSTG): Y balance test for the right leg and left leg↑, Stork balance (right leg) ↑
12	(R. Hammami et al., 2023)	32	Pre-pubertal weightlifter	Male	10.94 ± 0.47 years	E.G., (IRT1): Instability resistance training, 2 sets x 8, 20% (1RM) E.G., (IRT2): Instability resistance training, 2 sets x 4, 40% 1RM CG: No control group	1) Airex Balance Beam 2) Airex Balance Pad, 3) Thera-Band Stability Trainer 4) Togu Aero Step	Reciprocal balance test: 1) CoP displacements of the center of pressure oscillation test	5 times/Week, 8 weeks	E.G., (IRT1): CoP SA↑, CoP X↑, CoP Y↑, CoP V↑ E.G., (IRT2): CoP SA↑, CoP X↑, CoP Y↑, CoP V↔
13	Oliver and Brezzo (2009)	26	Volleyball and Soccer player	Female	Volleyball: 19.9 ± 1.8 years Soccer player: 18.5 ± 0.5 years	E.G.,: Functional balance training for volleyball player CG: No intervention for soccer player	1) The Indo Board 2) Flow cushion	Static balance test: 1) Biodex balance test	4 times/Week, unknown weeks	E.G., (Volleyball player): Biodex (right) ↔, Biodex (left) ↔ CG (Soccer player): Biodex (right) ↔, Biodex (left) ↔
14	Gönener and Gönener (2020)	40	Gymnast	Female	7 years old	E.G.,: Unstable surface training CG: Stable surface training	1) BOSU ball 2) Balance board 3) Sponge 4) Trampoline	Dynamic balance test: 1) Tecno-body ProKin PK200 model dynamic balance test	3 times/Week, 8 weeks	E.G.,: PL↑, AGP↑, MS↑, AP↑, ML↔ CG: PL↔, AGP↔, MS↔, AP↔, ML↔
15	Zemková and Hamar (2010)	34	Basketball player	Female	E.G.,: 20.9 ± 2.4 years CG: 21.2 ± 2.8 years	E.G.,: Combined agility-balance training on wobble boards CG: Combined agility-balance training on stable surface	1) Wobble boards	Static balance test: 1) Eyes open and closed Bipedal stance on stable platform 2) Eyes open and closed Bipedal stance on wobble boards	4–5 times/Week, 6 weeks	E.G.,: Bipedal stance on wobble board with eyes open and closed↓
16	Hammami et al. (2020)	31	Handball player PS:11 P:10 C:10	Male	PS: 16.2 ± 0.6 P: 16.4 ± 0.5 C: 16.5 ± 0.4	E.G., (PS): plyometrics training on sand surface CG (P): Standard plyometrics training on a stable surface CG (C): A standard in season regimen	1) On sand surface	Dynamic balance test: 1) Y Balance Test Static balance test: 2) Stork Balance Test	3 times/Week, 7 weeks	*Y Balance Test* E.G., (PS): right leg, RL/L↑, RL/B↑, RL/R↔; left leg, RL/L↔, RL/B↑, RL/R↔;CG (P): right leg, RL/L↑, RL/B↑, RL/R↔; left leg, RL/L↔, RL/B↑, RL/R↑;CG (C): right leg, RL/L↔, RL/B↑, RL/R↔; left leg, RL/L↔, RL/B↑, RL/R↔ *Stork Balance Test:* E.G., (PS): right leg↑; left leg↑ CG (P): right leg↑; left leg↑ CG (C): right leg↑; left leg↑
17	Eisen et al. (2010)	36	Volleyball and Soccer players	Mixed: Female and Male	18–22 years	E.G., (DD): Balance training with uniaxial on a rocker board E.G., (RB): Balance training with multiaxial on a dynadisc CG (CON): No training	1) Uniaxial on a rocker board [RB] 2) Multiaxial on a dynadisc [DD]	Dynamic balance test: 1) Star excursion balance test (SEBT)	3 times/Week, 4 weeks	Between-group E.G.,(DD), E.G., (RB) and CG (CON) differences at post-test: SEBT↑
18	Gidu et al. (2022)	96	Soccer player	Male	E.G.,: 14.2 ± 0.4 years CG: 14.0 ± 0.0 years	E.G.,: Proprioceptive training on Bosu ball CG: Normal program	1) BOSU ball	Static balance test: 1) Single-leg stance 2) Double-leg stance 3) Tandem stance 4) Total BESS score	4 times/Week, 8 weeks	E.G.,: 1) Single-leg stance↑ 2) Double-leg stance ↑ 3) Tandem stance↑ 4) Total BESS score↑
19	Granacher et al. (2015)	24	Soccer players	Male	15 ± 1 year	E.G.,: Unstable plyometric training (IPT) CG: Stable plyometric training (SPT)	1)Balance beam, pad, Airex^®^ 2)Stability trainer, Thera-Band^®^ 3)Togu^®^ Aero Step	Static balance test: 1) One-legged balance test Dynamic balance test: 1) Star excursion balance test (SEBT)	2 times/Week, 8 weeks	E.G.,: Star excursion balance test (SEBT) ↑
20	Chaouachi et al. (2017)	26	Soccer players	Male	13.9 ± 0.3 years	E.G., (ABPT): Alternated balance and plyometric exercises with unstable surface E.G., (BBPT): Balance and plyometric exercises with unstable surface	1) BOSU and Swiss ball 2) Inflated disc and foam surface	Static balance test: 1) Stork stands balance protocol Dynamic balance test: 1) Y balance test	2 times/Week, 8 weeks	E.G., (ABPT): Stork stand balance protocol↑, Y balance test ↑ E.G., (BBPT): Stork stand balance protocol↑, Y balance test ↑

Note: NR, not reported; yrs, years; Exp, sports experience; M, male; F, female; Freq, frequency; reps, repetitions; EG, experimental group; CG, control group; IRT, instability resistance training; ↑ significant within-group improvement; ↔ non-significant within-group.

### 3.4 The type of instability intervention

Table 3 shows the type of instability intervention, unstable environment or surface, duration, and frequency for the 20 studies. These interventions of IRT included Instability suspension training of 3 articles (Norambuena et al., 2021; Thielen et al., 2020; Prasetyo et al., 2023), Instability balance training of 1 article (Kang et al., 2013), Combined balance and plyometric training (IRT is a part of the program) of 1 article (Makhlouf et al., 2018), Integrated functional core and plyometric training (IRT is a part of the program) of 1 article (Cabrejas Mata et al., 2022), Integrative neuromuscular training (IRT is a part of the program) of 1 article (Zhao et al., 2021), Unstable performed combined plyometric training (PTC) of 1 article (Negra et al., 2017), Proprioceptive training program on BOSU and Swiss ball of 2 article (Romero-Franco et al., 2012; Gidu et al., 2022), Soccer program supplementary Swiss Ball Training (SBT) of 1 article (Wee and Fatt, 2019), Supplemental jump and sprint exercise training on sand of 1 article (Hammami et al., 2022), Instability resistance training (2 sets x 8, 20% (1RM)) of 1 article (Hammami et al., 2023), Functional balance training for volleyball player of 1 article (Oliver and Brezzo, 2009), Unstable surface training of 1 article (Gönener and Gönener, 2020), Combined agility-balance training on wobble boards of 1 article (Zemková and Hamar, 2010), Plyometrics training on sand surface of 1 article (Hammami et al., 2020), Balance training with uniaxial on a rocker board of 1 article (Eisen et al., 2010), Unstable plyometric training (IPT) of 1 article (Granacher et al., 2015), and Alternated balance and plyometric exercises with unstable surface of 1 article (Chaouachi et al., 2017).

In the type of unstable surface or environment, a common feature is that separating or integrating training with instability intervention used unstable surface or environment for each intervention or training activity. Among the 20 studies, 6 articles reported the unstable environments of Swiss ball, including only using Swiss ball of 2 articles (Kang et al., 2013; Wee and Fatt, 2019), combined using Swiss ball with other unstable environments of 4 articles (Makhlouf et al., 2018; Zhao et al., 2021; Romero-Franco et al., 2012; Chaouachi et al., 2017), also 6 articles reported the unstable environments of BOSU, including only using BOSU of 2 articles (Prasetyo et al., 2023; Gidu et al., 2022), combined using BOSU with other unstable environments of 4 article (Cabrejas Mata et al., 2022; Zhao et al., 2021; Gönener and Gönener, 2020; Chaouachi et al., 2017), 8 articles reported the unstable environments of Wobble boards, including only using Wobble boards of 2 article (Zemková and Hamar, 2010; Oliver and Brezzo, 2009), combined using Wobble boards with other unstable environments of 6 articles (Makhlouf et al., 2018; Zhao et al., 2021; Negra et al., 2017; Gönener and Gönener, 2020; Granacher et al., 2015; Chaouachi et al., 2017). Moreover, among the 20 studies, except for the Swiss ball, BOSU, and Wobble boards, two articles reported the unstable environments of the Suspension trainer (Norambuena et al., 2021; Thielen et al., 2020), the Elastic band strap of 1 article (Makhlouf et al., 2018), Thera-Band stability trainer and Togu^®^ Aero Step of 3 articles (Negra et al., 2017; Hammami et al., 2023; Granacher et al., 2015), the Sand surface of 2 articles (Hammami et al., 2020; Hammami et al., 2022), Foam surface and Sponge of 2 articles (Gönener and Gönener, 2020; Chaouachi et al., 2017), Trampoline of 1 article (Gönener and Gönener, 2020), and Uniaxial on a rocker board and Multiaxial on a dyna-disc of 1 article (Eisen et al., 2010).

As for duration, 18 out of 20 articles reported the duration of IRT intervention, and only two articles did not report duration (Prasetyo et al., 2023; Oliver and Brezzo, 2009), including ten articles on 8 weeks (Kang et al., 2013; Makhlouf et al., 2018; Cabrejas Mata et al., 2022; Zhao et al., 2021; Negra et al., 2017; Hammami et al., 2023; Gönener and Gönener, 2020; Gidu et al., 2022; Granacher et al., 2015; Chaouachi et al., 2017), four articles on 6 weeks (Thielen et al., 2020; Romero-Franco et al., 2012; Wee and Fatt, 2019; Zemková and Hamar, 2010), two articles on 7 weeks (Hammami et al., 2022; Hammami et al., 2020), 1 article on 5 weeks (Norambuena et al., 2021), and 1 article on 4 weeks (Eisen et al., 2010). The above 20 references have shown that a 4–8 weeks short-term duration of IRT intervention could improve balance ability for different sports athletes.

Regarding frequency, the typical training frequency was 2–5 per week. Among these studies, six studies’ training frequency was three times per week (Cabrejas Mata et al., 2022; Romero-Franco et al., 2012; Hammami et al., 2022; Gönener and Gönener, 2020; Hammami et al., 2020; Eisen et al., 2010); 4 studies’ training frequency was two times per week (Makhlouf et al., 2018; Wee and Fatt, 2019; Granacher et al., 2015; Chaouachi et al., 2017); 4 studies’ training frequency was four times per week (Thielen et al., 2020; Zhao et al., 2021; Oliver and Brezzo, 2009; Gidu et al., 2022); 2 studies’ training frequency was five times per week (Norambuena et al., 2021; Hammami et al., 2023); 1 studies’ training frequency was 3–5 times per week (Negra et al., 2017); 1 studies’ training frequency was 4–5 times per week (Zemková and Hamar, 2010); and the remaining two studies’ training frequency was unknow times per week (Kang et al., 2013; Prasetyo et al., 2023).

## 4 Outcome

The division of balance ability can be shown in Table 4. According to the balance ability classification theory in the field of sports training, there is one general category for balance ability (Tian, 2006; Michael, 2016; Deng et al., 2018). Among them, there are three subcategories in the balance ability type of general category: 1) Reciprocal balance ability, 2) Static balance ability, and 3) Dynamic balance ability. Therefore, this study systematically summarized and analyzed the results of 20 papers based on the general category and subcategories of balance ability mentioned above. The detail is shown in Table 4.

**TABLE 4:** The general, subcategories, and test method of balance ability.

General Category	Subcategories	Main representative test methods
Type of balance ability	Reciprocal balance ability	(1) Postural stability balance (2) Center of gravity test (3) CoP displacements of the center of pressure oscillation test
	Static balance ability	(1) The stork stand test (2) One-leg standing with open or closed eyes balance test (3) Bipedal (double-leg) stance test
	Dynamic balance ability	(1) Star excursion balance test (SEBT) (2) Y Balance Test

### 4.1 Effect of IRT on reciprocal balance ability

Table 3 shows that three studies have explored the effect of IRT intervention on reciprocal balance ability among ten judo athletes (Norambuena et al., 2021), 33 sprinters (Romero-Franco et al., 2012), and 32 pre-pubertal weightlifters (Hammami et al., 2023). One article showed t that there was no significant difference between the pre and post-test of the experimental group of judo athletes in the prone balance test for reciprocal balance ability (0.70 ± 0.48 vs. 0.40 ± 0.52, p > 0.05) (Norambuena et al., 2021). Meanwhile, in another study, the participants were track and field athletes. Between-group comparison, E.G., and C.G., the study results showed that it is significant differences in improved reciprocal balance ability in terms of XEO (E.G.,: −0.78 ± 4.31 vs. C.G.: 2.30 ± 2.75, p < 0.01), the position of the gravity center in the posterior (E.G., 69.75 ± 18.20 vs. C.G.: 54.71 ± 17.68, p < 0.05) and suitable (E.G.,: 63.31 ± 17.15 vs. C.G.: 49.71 ± 23,087, p < 0.05) direction test after proprioceptive training program on BOSU and Swiss ball (Romero-Franco et al., 2012). The result of the third study showed a statistically significant difference between the two experimental groups for pubertal weightlifters’ reciprocal balance ability on CoP displacements of the center of pressure oscillation test: CoP S.A. (IRT1: 1940.17 vs. 548.21, p > 0.01; IRT2: 2,158.47 vs. 692.80, p > 0.01), CoP X (IRT1: 2,110.29 vs. 1,687.12, p > 0.05; IRT2: 2,134.54 vs. 1,690.68, p > 0.05), CoP Y (IRT1: 2,110.78 vs. 1728.89, p > 0.05; IRT2: 2,247.15 vs. 1936.52, p > 0.05), CoP V (IRT1: 63.61 vs. 50.76, p > 0.05) (Hammami et al., 2023).

### 4.2 Effect of IRT on static balance ability

Table 3 shows that IRT intervention impacts static balance ability: one-leg standing with open and closed eyes stable and unstable test, Biodex balance test (eyes open and closed Bipedal stance test), and the stork stand test. Three studies show that IRT intervention can provide instability resistance training, which can help improve static balance ability for weightlifters (Kang et al., 2013), rhythmic gymnasts (Cabrejas Mata et al., 2022), soccer players (Gidu et al., 2022) in one-leg standing with open and closed eyes stable and unstable test. However, one article shows no significant difference in one-leg standing balance for soccer players after IRT training (Granacher et al., 2015). Similar to the one-leg standing balance test, three studies have explored the effect of the IRT program on the Bipedal (double-leg) stance test. Two studies found that IRT intervention can improve (double-leg) stance static balance ability for Basketball players (Zemková and Hamar, 2010) and soccer players (Gidu et al., 2022). However, after the IRT training, Oliver and Brezzo found no significant difference in the Biodex balance test for volleyball and soccer players (Oliver and Brezzo, 2009). Moreover, the stork stand test result is mostly for static balance ability. Six studies showed statistically significant within-group differences for the pre and post-test of the experimental group. Between-group were noted for the stork stand the test of static balance ability among archery athletes (Prasetyo et al., 2023), soccer players (Makhlouf et al., 2018; Negra et al., 2017; Chaouachi et al., 2017), handball player (Hammami et al., 2022; Hammami et al., 2020). Only two articles showed no significant difference for soccer players (Wee and Fatt, 2019; Negra et al., 2017) in the stork stand test of static balance ability between the experimental and control groups.

### 4.3 Effect of IRT on dynamic balance ability

Table 3 shows that IRT intervention has an impact on the following aspects of dynamic balance ability: Y balance test, Star excursion balance test (SEBT), single-leg side hop test, four-step square test (FSST), Tecno-body ProKin PK200 model dynamic balance test. In this study, five studies show that IRT intervention can provide instability resistance training, which can help improve dynamic balance ability in Y balance test for judo athletes (Norambuena et al., 2021), soccer player (E.G.,: P < 0.01, d = 1.20; C.G.: P = 0.18, d = 0.28) (Makhlouf et al., 2018), soccer player (both ABPT and BBPT experimental groups) (Chaouachi et al., 2017), handball player (Hammami et al., 2022), handball player (right leg, RL/L, RL/B; left leg, RL/B); (Hammami et al., 2020). A total of 2 articles showed a statistically significant difference in participants’ dynamic balance ability in the Star excursion balance test (SEBT) for mixed volleyball and soccer players (Eisen et al., 2010) and soccer players (Granacher et al., 2015). However, 1 article showed no statistically significant difference in participants’ dynamic balance ability in the Star excursion balance test (SEBT) for basketball players after the IRT training (Thielen et al., 2020). Other dynamic balance ability testing methods were used in the following two articles. One study examined the significant improvement impact of IRT for the dynamic balance ability of, E.G.,1 and, E.G.,2 with BOSU, Swiss ball, and Balance board for Single-leg side hop test (Left single-leg side hop, Right single-leg side hop) of 38 badminton players (17 ± 1.1 years) (Zhao et al., 2021). Another article revealed that the soccer program supplementary Swiss Ball Training (SBT) intervention did provide additional benefit to the dynamic balance ability for The step square test (FSST) of 19 soccer players (20 ± 1.73 years) (Wee and Fatt, 2019).

## 5 Discussion

The review included 20 studies involving diverse athletes and IRT interventions. The outcomes assessed in these studies varied for balance abilities. IRT is the process of creating an “unstable” platform by altering the stability of the support surface, imbalanced movement symmetry, and the occurrence of unexpected external forces that cause internal or external force imbalances in the body ((Behm et al., 2010). The findings of this systematic review provide robust evidence supporting the positive effects of IRT intervention on reciprocal, static, and dynamic balance ability among athletes. These findings are essential for developing targeted IRT programs to promote balance abilities.

### 5.1 Effect of IRT on reciprocal balance ability

Three studies empirically showed that IRT separately or integrating other training interventions could significantly improve judo athletes in prone balance test for reciprocal balance ability (Norambuena et al., 2021), track and field athletes’ reciprocal balance ability in terms of position of the gravity center direction test after proprioceptive training program on BOSU and Swiss ball (Romero-Franco et al., 2012), and pubertal weightlifters’ reciprocal balance ability on CoP displacements of the center of pressure oscillation test (Hammami et al., 2023).

The reasonable explanation for the results in which IRT intervention could significantly improve the reciprocal balance ability of the three studies is as follows. Firstly, the reciprocal balance ability refers to the kind of balance ability of the human body to evenly distribute the weight of the body to the fulcrums of the body, such as the force on both feet during standing time and whether the force on both buttocks is even during sitting position (Evangelidis et al., 2016). The human body maintains balance through complex visual, vestibular, and proprioceptive systems (Bliss and Teeple, 2005). Reciprocal balance ability plays a crucial role in maintaining the stability of human posture and preventing falls. It especially maintains stability in the hip, knee, and ankle joints (Martino et al., 2023). Secondly, some research reports by scientists indicated that among subjects trained on unstable and stable surfaces, those trained on stable surfaces have poorer reciprocal balance abilities (Hirase et al., 2015). In addition, one of the essential interpretation criteria for reciprocal balance ability development is to activate visual and sensory-motor units by training on unstable surfaces and to adapt the body sway and body stability to these surfaces (Gönener and Gönener, 2020).

### 5.2 Effect of IRT on static balance ability

Eleven studies show that IRT can improve static balance ability for weightlifter (Kang et al., 2013), rhythmic gymnast (Cabrejas Mata et al., 2022), soccer player (Gidu et al., 2022) in one-leg standing with open and closed eyes stable and unstable test, Bipedal (double-leg) stance test of static balance ability for Basketball player (Zemková and Hamar, 2010), and soccer player (Gidu et al., 2022), and stork stand test of static balance ability about archery athletes (Prasetyo et al., 2023), soccer player (Makhlouf et al., 2018; Negra et al., 2017; Chaouachi et al., 2017), handball player (Hammami et al., 2022; Hammami et al., 2020).

The possible and reasonable explanations for IRT could significantly improve athletes’ static balance ability of one-leg standing with open and closed eyes test, Bipedal (double-leg) stance test, and stork stand test are as follows. Firstly, the static balance ability refers to the ability of the human body to maintain a specific posture for a particular duration of time in a relatively static state, such as standing, standing upside down, archery, and other movements that are all static balance ability (Hrysomallis, 2007). In addition, the IRT training program consists of continuous unstable surface exercises in different unstable environments, improved static postural control, and hip, knee, and ankle joint strength production in athletes. The moderate instability of the unstable surface resulted in greater muscle activation than performing exercises on the floor (stable surface) (Gao et al., 2024). However, based on Table 3, four articles found that IRT has not improved static balance ability and no significant difference in one-leg standing balance for soccer players (Granacher et al., 2015; Wee and Fatt, 2019; Negra et al., 2017), and Biodex balance test for volleyball and soccer player (Oliver and Brezzo, 2009). The possible explanation is that the training duration and frequency were relatively short (only two times per week) or maybe that only a tiny portion of the training actions in the intervention used an unstable surface or environment, and a small amount of unstable intervention resulted in no significant difference in static balance ability between the, E.G., and C.G. groups.

### 5.3 Effect of IRT on dynamic balance ability

In this study, nine studies show that IRT can help improve dynamic balance ability in Y balance test for judo athletes (Norambuena et al., 2021), soccer player (Makhlouf et al., 2018; Chaouachi et al., 2017), handball player (Hammami et al., 2022; Hammami et al., 2020), star excursion balance test (SEBT) for mixed volleyball and soccer players (Eisen et al., 2010), soccer players (Granacher et al., 2015), single-leg side hop test for badminton players (Zhao et al., 2021), and four step square test (FSST) of soccer players (Wee and Fatt, 2019).

Perhaps the main reason for this result is that young athletes of different genders participating in IRT can affect their dynamic balance ability as follows. Research in biomedical fields highlights that hormonal differences significantly affect musculoskeletal structure and function, influencing dynamic balance and overall stability. For instance, conditions such as adolescent idiopathic scoliosis and osteoporosis, which occur more frequently in females, can impair postural control and balance due to altered spinal alignment and reduced bone density (Biz et al., 2006). Similarly, ligamentous injuries, such as ACL tears, are more prevalent in female athletes due to differences in ligament laxity and neuromuscular control, further affecting dynamic stability during high-intensity movements (Hewett et al., 2006).

Thus, firstly, the dynamic balance ability refers to the ability of the human body to control body posture during movements, such as Trampoline, Stunts, and Ice dance, all of which require athletes to have good dynamic balance ability (Chtara et al., 2016). Secondly, the possible reason is that to complete different IRT interventions (such as instability suspension training, balance training, combined balance, and plyometric training, unstable performed combined plyometric training, a proprioceptive training program on BOSU and Swiss ball, and supplemental jump and sprint exercise training on the sand and so on), the athlete needs not only the muscle strength of the core part to perform contraction but also the dynamic balance ability (depends on the comprehensive coordination ability of the human body to stimuli from vestibular organs, proprioceptive receptors composed of muscles, tendons, joints, and visual organs) to perform working together to complete the action above. Therefore, the influence of the dynamic balance ability of the athlete’s body is that many complex factors work together that leads to this result (Wang et al., 2016). Last but not least, exercising on a relatively unstable surface increased the dynamic balance ability parameters of the experimental group, which may be explained by the fact that unstable training improved the supplementation patterns of deep and small muscle groups. Therefore, a high correlation coefficient exists between enhancing muscle activity and unstable environments (Lago-Fuentes et al., 2018; Gibbons and Bird, 2019). In addition, even though the, E.G., and C.G. groups performed similar exercises, those carried out on unstable surfaces can stimulate neural adaptation, improve neuro-muscular recruitment, enable the effective synchronization of motor units, lower neuro-inhibitory reflexes, and facilitate adequate proprioceptive feedback information for subjects’ dynamic balance ability (Hirase et al., 2015). Moreover, the reasonable explanation of the only 1 article’s results was not statistically significant in participants’ dynamic balance ability in the Star excursion balance test (SEBT) for basketball players after instability suspended load training, and the possible explanation is that the training cycle is relatively short just 6 weeks.

## 6 Limitations

This review has a few limitations that need to be highlighted. 1) The current literature does not investigate the impact of the IRT program on other balance ability parameters such as the center of gravity balance, visual balance, and nervous system balance. Future research in this IRT intervention should address these aspects to acquire a deeper understanding. 2) The existing studies focus on the unstable surfaces of the environment or IRT, mainly using Swiss and BOSU. Therefore, future studies should develop other new unstable surfaces or environments, such as water, snow, sand surfaces, elastic bands, and suspension instability methods and environments. 3) Most interventions (80%–90%) in the included studies were unstable training interventions combined with other training methods, and there are not many IRT intervention methods in the literature for balance ability. Future studies should develop single IRT interventions for athletes in different events. 4) In the research results of the literature, there was a lack of analysis of covariance on the impact of IRT on balance ability for different athletes.

## 7 Conclusion

This systematic review of IRT on reciprocal, static, and dynamic balance ability among athletes provided evidence and materials that unstable surface training could improve the balance ability of judo athletes, basketball players, weightlifters, archery athletes, soccer players, rhythmic gymnasts, badminton Players, track and field athletes, handball players, volleyball players, and gymnasts using unstable surfaces or environments (i.e., BOSU, Swiss ball, Wobble boards, Suspension trainer, Sissel pillows, Inflated disc and foam surface, Airex balance pad, Togu power ball, Thera-Band, Elastic band strap, Sand surface and so on). Theoretically, this study indicated that IRT is an improvement training method for activating deep layers and small muscles of the human body, enhancing coordination between agonistic and antagonistic muscles, and improving nerve vestibule, proprioceptive sense, and visual sense for the Equilibrium Triad System of Balance Ability. Therefore, this review suggests that IRT should be considered in athletes’ daily training routines for balance ability.

## Data Availability

The original contributions presented in the study are included in the article/Supplementary Material, further inquiries can be directed to the corresponding author.
