# Supplementary material for: The effect of instability resistance training on balance ability among athletes: a systematic review
Source: Front Physiol. 2025 Jan 7;15:1434918. doi: 10.3389/fphys.2024.1434918 (PMC11746901; doi:10.3389/fphys.2024.1434918)
Supplement: Supplementary file 1 [file DataSheet1.pdf]

Supplementary Material

1 Supplementary Figures and Tables

1.1 Supplementary Figures

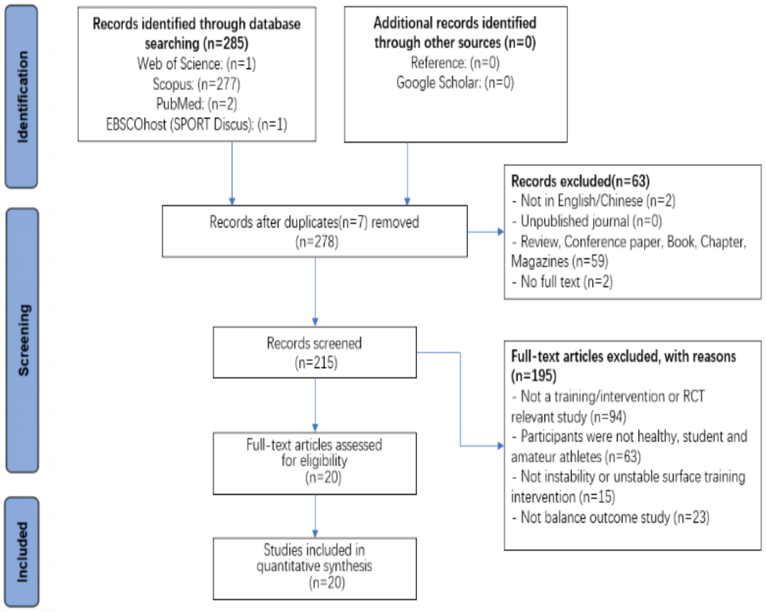

Figure 1 The Identification, Screening, and Included Processes for Articles Based on PRISMA

1.2 Supplementary Tables

Table 1 Inclusion and Eligibility Criteria

PICOS	Detailed Information on inclusion and eligibility criteria
**Population**	Healthy athletes or players cannot distinguish between age and gender.
**Intervention**	IRT (with the different unstable surfaces), IRT+, another training format in EG (not < 4 weeks)
**Comparison**	Single (within group), multiple-group trials (between groups)
**Outcome**	The outcome must comprise the impact of IRT on balance ability among athletes and players.
**Study Design**	Single-group and randomized controlled trials (RCT or CRCT)

Table 2 Summary of PEDro Scale Assessment Scores

N	Reference	N1	N2	N3	N4	N5	N6	N7	N8	N9	N10	N11	Total PEDro Score	Study Quality
1	(Norambuena et al., 2021)	1	0	0	1	0	0	0	1	0	0	1	4	Moderate
2	(Thielen et al., 2020)	1	1	0	1	0	0	0	1	1	1	1	7	High
3	(Kang et al., 2013)	1	1	0	1	0	0	0	1	1	1	1	7	High
4	(Prasetyo et al., 2023)	1	0	0	1	0	0	0	1	0	0	1	4	Moderate
5	(Makhlouf et al., 2018)	1	1	0	1	0	0	0	1	1	1	1	7	High
6	(Cabrejas Mata et al., 2022)	0	1	0	1	0	0	0	1	1	1	1	6	Moderate
7	(Zhao et al., 2021)	1	0	0	1	0	0	0	1	0	0	1	4	Moderate
8	(Negra et al., 2017)	1	1	0	1	0	0	0	1	1	1	1	7	High
9	(Romero-Franco et al., 2012)	1	1	0	1	0	0	0	1	1	1	1	7	High
10	(Wee & Fatt, 2019)	1	1	0	1	0	0	0	1	1	1	1	7	High
11	(M. Hammami et al., 2022)	1	1	0	1	0	0	0	1	1	1	1	7	High
12	(R. Hammami et al., 2023)	1	0	0	1	0	0	0	1	0	1	1	5	Moderate
13	(Oliver & Brezzo, 2009)	1	1	0	1	0	0	0	1	1	1	1	7	High
14	(Gönener & Gönener, 2020)	1	1	0	1	0	0	0	1	1	1	1	7	High
15	(Zemková & Hamar, 2010)	1	1	1	1	0	0	0	1	1	1	1	8	High
16	(M. Hammami et al., 2020)	0	1	0	1	0	0	0	1	1	1	1	6	High
17	(Eisen et al., 2010)	1	1	0	1	0	0	0	1	1	1	1	7	High
18	(Gidu et al., 2022)	1	1	0	1	0	0	0	1	1	1	1	7	High
19	(Granacher et al., 2015)	1	1	0	1	0	0	0	1	1	1	1	7	High
20	(Chaouachi et al., 2017)	0	1	0	1	0	0	0	1	1	0	1	5	Moderate

Note: N1, Eligibility Criteria; N2, Random Allocation; N3, Allocation Concealment; N4, Baseline Comparability; N5, Blind Participants; N6, Blind Therapist; N7, Blind Assessor; N8, Follow-Up; N9, Intention to Treat Analysis; N10, Group Comparison; N11, Point Measure and Variability. A detailed explanation for each PEDro scale item can be accessed at https://www.pedro.org.au/english/downloads/pedro-scale.

Table 4 The General, Subcategories, and Test Method of Balance Ability

General Category	Subcategories	Main Representative test methods
Type of balance ability	Reciprocal balance ability	(1) Postural stability balance<br>(2) Center of gravity test<br>(3) CoP displacements of the center of pressure oscillation test
	Static balance ability	(1) The stork stand test<br>(2) One-leg standing with open or closed eyes balance test<br>(3) Bipedal (double-leg) stance test
	Dynamic balance ability	(1) Star excursion balance test (SEBT)<br>(2) Y Balance Test

Table 3 Participant, Intervention, and Main Outcome for the 20 Studies

N	Study	Subjects	Intervention							Main outcome related to balance ability
			Type of athlete	Gender	Age	Type	Instability environment	Balance ability Measured index	Frequency & duration	
1	(Norambuena et al., 2021)	10	Judo athletes	Mixed:<br>8 Female<br>2 Male	15.4±2.8 y	EG: Instability suspension training<br>CG: No control group	1) Suspension trainer (TRX®, USA)	Reciprocal balance test:<br>1) Prone instability test<br>**Dynamic balance test:**<br>1) Y balance test	5 times/ Week,<br>5 weeks	**EG:** Prone instability test↔,<br>Y balance test of right leg↑, left leg↑, right arm↑, left arm↑
2	(Thielen et al., 2020)	32	Basketball player	Male	20.4±1.4 y	EG: Suspended load program<br>CG: Traditional barbell load program	1) The plates suspended from the barbell using non-elastic straps	**Dynamic balance test:**<br>1) Star excursion balance test (SEBT)	4 times/ Week,<br>6 weeks	**EG and CG**: Star excursion balance test (SEBT)↔
3	(Kang et al., 2013)	32	Weightlifter	Male	MS: 14y<br>HS: 17y	EG1 (MS): Instability balance training<br>EG2 (HS): Instability balance training<br>CG1 (MS): No training<br>CG2 (HS): No training	1) Swiss ball	**Static balance test:**<br>1) One-leg standing with closed eyes test	Unknown times/ Week,<br>8 weeks	**EG2 (HS)**: One-leg standing time with eyes closed↑
4	(Prasetyo et al., 2023)	12	Archery athletes	Unknown	14-17y	EG: Suspension training<br>CG: No control group	1) BOSU	**Static balance test:**<br>1) The stork stand test	18 circuit training sessions	**EG:** Standing on one leg↑
5	(Makhlouf et al., 2018)	57	Soccer player	Male	EG1:11.06±0.75y<br>EG2:11.29±0.85y<br>CG:10.98±0.80y	EG1 (BPT): Combined balance and plyometric training (instability training is a part of the program)<br>EG2 (APT): Agility-plyometric training (stable training)<br>CG: Regular soccer training (stable training)	1)Swiss ball,<br>2) An inflated disk,<br>3) A foam surface progressing to a BOSU or inflated disk,<br>4) Elastic band strap	**Static balance test:**<br>1) Standing stork test<br>**Dynamic balance test:**<br>1) Y balance test	2 times/ Week,<br>8 weeks	**BTP:** Standing stork (P<0.01, d=3.17) ↑, Y-balance (P<0.01, d=1.48) ↑;<br>**ATP:** Standing stork (P<0.01, d=5.53) ↑, Y-balance (P<0.01, d=1.20) ↑;<br>**CG:** Standing stork (P<0.02, d=1.30) ↑, Y-balance (P=0.18, d=0.28)↔
6	(Cabrejas Mata et al., 2022)	44<br>EG: 23<br>CG: 21	Rhythmic gymnast	Female	10.5±1.8 y	EG: Integrated functional core and plyometric training (instability training is a part of the program)<br>CG: Usual training (stable training)	1) BOSU ball,<br>2) Balance disks,<br>3) Softballs	**Static balance test:**<br>1) Right/left support leg with eyes open test,<br>2) Right/left support leg with eyes closed test	3 times/ Week,<br>8 weeks	**EG:** Right support leg with eyes open ↑; Left support leg with eyes open ↑
7	(Zhao et al., 2021)	38	Badminton Player	Female	17±1.1 y	EG1 (HG): Integrative neuromuscular training (instability training is a part of the program)<br>EG1 (LG): Integrative neuromuscular training (instability training is a part of the program)<br>CG: No control group	1) BOSU,<br>2) Balance board,<br>3) Swiss ball	**Dynamic balance test:**<br>1) Single-leg side hop test	4 times/ Week,<br>8 weeks	**EG1 (HG):** Single-leg side hop (L) ↑, Single-leg side hop (R) ↑;<br>**EG2 (LG):** Single-leg side hop (L) ↑, Single-leg side hop (R) ↑
8	(Negra et al., 2017)	33	Soccer player	Male	PTS: 12.1±0.5 y<br>PTC: 12.2±0.6 y	EG: Unstable performed combined plyometric training (PTC)<br>CG: Stable performed plyometric training (PTS)	1) Airex balance pad,<br>2) Thera-Band stability trainer	**Static balance test:**<br>1) SSBT, stable stork balance test<br>2) USBT, unstable stork balance test	3-5 times/ Week,<br>8 weeks	**Between-group (post-test)**<br>SSBT↔, USBT ↑
9	(Romero-Franco et al., 2012)	33	Sprinter	Male	21.82±4.84 y	EG: Proprioceptive training program on BOSU and Swiss ball<br>CG: A shorter duration training	1) BOSU and Swiss ball	**Reciprocal balance test:**<br>1) Stability Test with Eyes Open and Closed; 2) Postural Stability;<br>3) Gravity Center Control	3 times/ Week,<br>6 weeks	**Between-group (post-test):**<br>XEO↑, Position of the gravity center in the posterior direction ↑, Position of the gravity center in the right direction ↑
10	(Wee & Fatt, 2019)	19	Soccer Player	Male	20±1.73 y	EG: Soccer program supplementary Swiss Ball Training (SBT)<br>CG: Soccer program	1) Swiss Ball	**Static balance test:**<br>1) Standing Stork Test（SST）<br>**Dynamic balance test:**<br>1) Four Step Square Test （FSST）	2 times/ Week,<br>6 weeks	**Between-group (post-test):**<br>SST↔, FSST↑,

11	(M. Hammami et al., 2022)	42	Handball player	Male	EG: 16.4±0.4 y<br>CG: 16.2±0.4 y	EG (JSTG): Supplemental jump and sprint exercise training on sand<br>CG: Standard in-season regimen	1) On sand	**Static balance test:**<br>1) Standing stork test,<br>**Dynamic balance test:**<br>2) Y balance test	3 times/ Week, 7 weeks	**EG (JSTG):** Y balance test for the right leg and left leg↑, Stork balance (right leg) ↑
12	(R. Hammami et al., 2023)	32	Pre-pubertal weightlifter	Male	10.94±0.47 y	EG (IRT1): Instability resistance training, 2 sets x 8, 20% (1RM)<br>EG (IRT2): Instability resistance training, 2 sets x 4, 40% 1RM<br>CG: No control group	1) Airex Balance Beam,<br>2) Airex Balance Pad,<br>3) Thera-Band Stability Trainer,<br>4) Togu Aero Step	**Reciprocal balance test:**<br>1) CoP displacements of the center of pressure oscillation test	5 times/ Week, 8 weeks	**EG (IRT1):** CoP SA↑, CoP X↑, CoP Y↑, CoP V↑<br>**EG (IRT2):** CoP SA↑, CoP X↑, CoP Y↑, CoP V↔
13	(Oliver & Brezzo, 2009)	26	Volleyball and Soccer player	Female	Volleyball: 19.9±1.8 y<br>Soccer player: 18.5±0.5 y	EG: Functional balance training for volleyball player<br>CG: No intervention for soccer player	1) The Indo Board<br>2) Flow cushion	**Static balance test:**<br>1) Biodex balance test	4 times/ Week, unknown weeks	**EG (Volleyball player):** Biodex (right) ↔, Biodex (left) ↔<br>**CG (Soccer player):** Biodex (right) ↔, Biodex (left) ↔
14	(Gönener & Gönener, 2020)	40	Gymnast	Female	7 years old	EG: Unstable surface training<br>CG: Stable surface training	1) BOSU,<br>2) Balance board,<br>3) Sponge,<br>4) Trampoline	**Dynamic balance test:**<br>1) Tecno-body ProKin PK200 model dynamic balance test	3 times/ Week, 8 weeks	**EG:** PL↑, AGP↑, MS↑, AP↑, ML↔<br>**CG:** PL↔, AGP↔, MS↔, AP↔, ML↔
15	(Zemková & Hamar, 2010)	34	Basketball player	Female	EG: 20.9±2.4 y<br>CG: 21.2±2.8 y	EG: Combined agility-balance training on wobble boards<br>CG: Combined agility-balance training on stable surface	1) Wobble boards	**Static balance test:**<br>1) Eyes open and closed Bipedal stance on stable platform<br>2) Eyes open and closed Bipedal stance on wobble boards	4-5 times/ Week, 6 weeks	**EG:** Bipedal stance on wobble board with eyes open and closed↓
16	(M. Hammami et al., 2020)	31	Handball player<br>PS:11,<br>P:10<br>C:10	Male	PS: 16.2±0.6,<br>P: 16.4±0.5<br>C: 16.5±0.4	EG(PS): plyometrics training on sand surface;<br>CG (P): Standard plyometrics training on a stable surface;<br>CG (C): A standard in season regimen	1) On sand surface	**Dynamic balance test:**<br>1) Y Balance Test<br>**Static balance test:**<br>2) Stork Balance Test	3 times/ Week, 7 weeks	***Y Balance Test***<br>**EG (PS):** right leg, RL/L↑, RL/B↑, RL/R↔; left leg, RL/L↔, RL/B↑, RL/R↔;**CG (P):** right leg, RL/L↑, RL/B↑, RL/R↔; left leg, RL/L↔, RL/B↑, RL/R↑;**CG (C):** right leg, RL/L↔, RL/B↑, RL/R↔; left leg, RL/L↔, RL/B↑, RL/R↔;<br>***Stork Balance Test:***<br>**EG (PS):** right leg↑; left leg↑;<br>**CG (P):** right leg↑; left leg↑;<br>**CG (C):** right leg↑; left leg↑
17	(Eisen et al., 2010)	36	Volleyball and Soccer players	Mixed: Female and Male	18-22 years	EG(DD): Balance training with uniaxial on a rocker board;<br>EG (RB): Balance training with multiaxial on a dynadisc;<br>CG (CON): No training	1) Uniaxial on a rocker board [RB]<br>2) Multiaxial on a dynadisc [DD]	**Dynamic balance test:**<br>1) Star excursion balance test (SEBT)	3 times/ Week, 4 weeks	**Between-group EG(DD), EG (RB) and CG (CON) differences at post-test:** SEBT↑
18	(Gidu et al., 2022)	96	Soccer player	Male	EG: 14.2±0.4 y<br>CG: 14.0±0.0 y	EG: Proprioceptive training on Bosu ball<br>CG: Normal program	1) BOSU	**Static balance test:**<br>1) Single-leg stance<br>2) Double-leg stance<br>3) Tandem stance<br>4) Total BESS score	4 times/ Week, 8 weeks	**EG:** 1) Single-leg stance↑,<br>2) Double-leg stance ↑,<br>3) Tandem stance↑,<br>4) Total BESS score↑
19	(Granacher et al., 2015)	24	Soccer players	Male	15±1 y	EG: Unstable plyometric training (IPT)<br>CG: Stable plyometric training (SPT)	1)Balance beam, pad, Airex®<br>2)Stability trainer, Thera-Band®<br>3)Togu® Aero Step	**Static balance test:**<br>1) One-legged balance test<br>**Dynamic balance test:**<br>1) Star excursion balance test (SEBT)	2 times/ Week, 8 weeks	**EG**: Star excursion balance test (SEBT) ↑
20	(Chaouachi et al., 2017)	26	Soccer players	Male	13.9±0.3 y	EG (ABPT): Alternated balance and plyometric exercises with unstable surface<br>EG (BBPT): Balance and plyometric exercises with unstable surface	1) BOSU and Swiss ball<br>2) Inflated disc and foam surface	**Static balance test:**<br>1) Stork stands balance protocol<br>**Dynamic balance test:**<br>1) Y balance test	2 times/ Week, 8 weeks	**EG** (ABPT): Stork stand balance protocol↑, Y balance test ↑<br>**EG** (BBPT): Stork stand balance protocol↑, Y balance test ↑

Note: NR, not reported; yrs, years; Exp, sports experience; M, male; F, female; Freq, frequency; reps, repetitions; EG, experimental group; CG, control group; IRT, instability resistance training; ↑ significant within-group improvement; ↔ non-significant within-group.
